# Siderophores in environmental research: roles and applications

**DOI:** 10.1111/1751-7915.12117

**Published:** 2014-02-27

**Authors:** E Ahmed, S J M Holmström

**Affiliations:** Department of Geological Sciences, Stockholm UniversityStockholm, Sweden

## Abstract

Siderophores are organic compounds with low molecular masses that are produced by microorganisms and plants growing under low iron conditions. The primary function of these compounds is to chelate the ferric iron [Fe(III)] from different terrestrial and aquatic habitats and thereby make it available for microbial and plant cells. Siderophores have received much attention in recent years because of their potential roles and applications in various areas of environmental research. Their significance in these applications is because siderophores have the ability to bind a variety of metals in addition to iron, and they have a wide range of chemical structures and specific properties. For instance, siderophores function as biocontrols, biosensors, and bioremediation and chelation agents, in addition to their important role in weathering soil minerals and enhancing plant growth. The aim of this literature review is to outline and discuss the important roles and functions of siderophores in different environmental habitats and emphasize the significant roles that these small organic molecules could play in applied environmental processes.

## Introduction

Iron (Fe) is an essential element for the growth of almost all living microorganisms because it acts as a catalyst in enzymatic processes, oxygen metabolism, electron transfer, and DNA and RNA syntheses (Aguado-Santacruz *et al*., 2012). Fe is also essential for biofilm formation because it regulates surface motility and stabilizes the polysaccharide matrix (Weinberg, 2004; Chhibber *et al*., 2013). Under iron-deficient growth conditions, the microbial surface hydrophobicity decreases which alters the surface protein composition and leads to limitation of biofilm formation (Simões *et al*., 2007). Thus, because of the low bioavailability of Fe in the environment, microorganisms have developed specific uptake strategies such as production of siderophores. Siderophores are metal-chelating agents with low molecular masses (200–2000 Da) that are produced by microorganisms and plants, especially under Fe-limiting conditions (Schwyn and Neilands, 1987). Marine organisms such as phytoplankton (Trick *et al*., 1983) and cyanobacteria (Armstrong and Van Baalen, 1979) can also produce siderophores. The role of siderophores is primarily to scavenge Fe, but they also form complexes with other essential elements (i.e. Mo, Mn, Co and Ni) in the environment and make them available for microbial cells (Bellenger *et al*., 2008; Braud *et al*., 2009a,b). Siderophores are divided into three main families depending on the characteristic functional group, i.e. hydroxamates, catecholates and carboxylates. More than 500 different types of siderophores are known, of which 270 have been structurally characterized (Boukhalfa *et al*., 2003). Hydroxamate siderophores have a 1:1 stability constant with Fe(III) that nears that of the Fe(III)-EDTA complex (10^30^), whereas catecholates and carboxylates can form 1:1 complexes with stability constants near that of Fe(III)-EDDHA (10^40^) (Robert and Chenu, 1992). The formation of Fe(III)–siderophore complexes are affected by pH because of the competition for the free siderophore ligands between free protons and Fe (Albrecht-Gary and Crumbliss, 1998). In nature, Fe has to compete not only against free protons for the siderophore binding sites but also against other metal ions such as divalent cations, including Cd^2+^, Cu^2+^, Ni^2+^, Pb^2+^ and Zn^2+^ (Albrecht-Gary and Crumbliss, 1998); trivalent cations, such as Mn^3+^, Co^3+^ and Al^3+^; and actinides, such as Th^4+^, U^4+^ and Pu^4+^ (Peterson *et al*., 2004). There are several studies that have shown that siderophores have an impact on the mobility of these metal ions in the environment (e.g. Renshaw *et al*., 2002; Dahlheimer *et al*., 2007; Schalk *et al*., 2011). Siderophores are not only contributing in plant and microorganism nutrition but also in other environmental applications. In this review, microbial and plant siderophores will be discussed with a primary focus on the roles, functions and applications of siderophores in different areas of environmental research.

## Microbial siderophores

**Figure 1:**
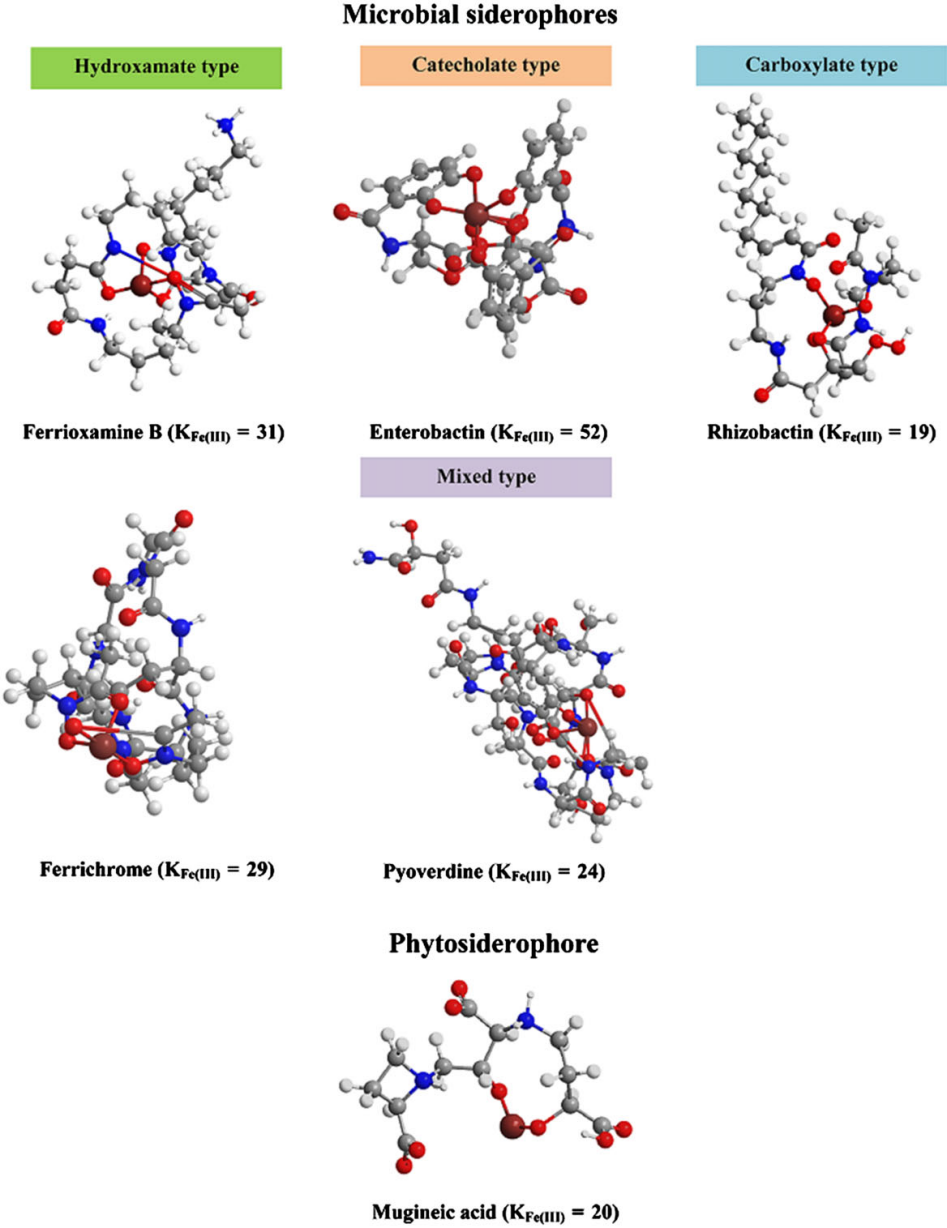
Representative examples of microbial siderophores and phytosiderophores with the stability constant of each type. Microbial siderophores consist of four main types. Hydroxamates produced by both bacteria (ferrioxamine B) and fungi (ferrichrome). Catecholate (enterobactin), carboxylate (rhizobactin) and mixed type (pyoverdine) produced by bacteria. The most common phytosiderophore is mugineic acid. All the chemical structures were drawn using ChemDraw Standard 13.0 software (PerkinElmer, Waltham, MA, USA).

Microorganisms produce a wide range of siderophores (Fig. 1). Most of the bacterial siderophores are catecholates (i.e. enterobactin), and some are carboxylates (i.e. rhizobactin) and hydroxamates (i.e. ferrioxamine B) (Matzanke, 1991). However, there are also certain types of bacterial siderophores that contain a mix of the main functional groups (i.e. pyoverdine) (Cornelis, 2010). One of the most common fungal siderophores is hydroxamates belonging to the ferrichrome family (i.e. ferrichrome), which is further divided into five groups, depending on the side chain of the hydroxamate functional group (Renshaw *et al*., 2002; Winkelmann, 2007).

Microorganisms use different siderophore-mediated Fe transport systems. For bacteria, the transport systems vary between gram-positive bacteria and gram-negative bacteria. Gram-negative bacteria (e.g. *Escherichia sp*.) have TonB-dependent outer membrane receptors that recognize the Fe(III)–siderophore complexes at the cell surface (Krewulak and Vogel, 2008). Once the Fe(III)–siderophore binds to the outer membrane receptor, the complex crosses the membrane through an energy-dependent system consisting of the outer membrane receptor proteins, periplasmic binding proteins and inner membrane transport proteins (Matzanke, 1991). Thereafter, the Fe(III)–siderophore complex that is bound by a high-affinity periplasmic binding protein, which accompanied the complex to the cytoplasmic membrane, is released into the periplasmic space (Noinaj *et al*., 2010). Then the complex is transported across the cytoplasmic membrane by an ATP-binding cassette (ABC) transport system, allowing it to reach the cytoplasm. Eventually, Fe(II) is released from the Fe(III)–siderophore complex via reduction of Fe(III). In contrast, in gram-positive bacteria (e.g. *Bacillus sp*.), which lack the outer membrane, the outer membrane receptors are completely absent. Therefore, the Fe(III)–siderophore complexes are bound by periplasmic siderophore binding proteins that are anchored to the cell membrane because of the lack of a periplasmic space (Fukushima *et al*., 2013). Then the Fe(III)–siderophore complexes are further transported by ATP-dependent transporters to the cytoplasm according to the ABC transport systems in the same way as the gram-negative bacteria (Braun and Hantke, 2011).

Fungi have four different mechanisms for siderophore-mediated Fe transport systems (Van der Helm and Winkelmann, 1994): (i) In the shuttle mechanism, the Fe(III)–siderophore complex is transported across the cell membrane, where the Fe(III) is released from the ligand and reduced by the reductive enzymes, whereas the free siderophore is then recycled. This mechanism is, for example, used for transporting ferrichrome in some fungal species such as *Ustilago maydis* (Ardon *et al*., 1998). (ii) In the taxicab mechanism, the Fe(III) from the extracellular siderophore is transferred across the cell membrane to intracellular ligands. This mechanism is used by *Rhodotorula* species (Winkelmann and Huschka, 1987). (iii) In the hydrolytic mechanism, the whole Fe(III)–siderophore complex is transported into the cell and is subjected to several reductive and degradative processes to release the Fe(III). The Fe(III) is reduced to Fe(II) inside the cell and the siderophore is excreted again. This mechanism is used in the uptake of Fe(III)–triacetylfusarinine complexes by *Mycelia sterilia* (Adjimani and Emery, 1988). (iv) In the reductive mechanism, the Fe(III)–siderophore complex is not transported across the cell membrane. The reduction of Fe(III) to Fe(II) occurs at the cell membrane and then the reduced Fe is taken up by the cell. This mechanism is used by *Ustilago sphaerogena* in the uptake of Fe(III) from ferrichrome (Ecker and Emery, 1983).

## Plant siderophores

Fe is an essential micronutrient for plant growth (Kobayashi and Nishizawa, 2012). Under conditions of Fe deficiency, graminaceous plants (e.g. barley and wheat) have developed an efficient strategy for acquiring Fe from insoluble sources (Kraemer *et al*., 2006). These plants secrete Fe(III)-chelating compounds called phytosiderophores that form specific strong complexes with Fe(III) (Ma, 2005). The phytosiderophores are hexadentate ligands that coordinate Fe(III) with their amino and carboxyl groups (Singh *et al*., 2011). When the phytosiderophore is released to the rhizosphere, it chelates Fe from the soil by forming Fe(III)–phytosiderophore complexes that can be subsequently transported across the root plasma membrane (Römheld and Marschner, 1986; Dell'mour *et al*., 2012). In comparison with the molecular mass of microbial siderophores, which is ranged between 200 and 2000 Da, phytosiderophores are ranged between 500 and 1000 Da (Neilands, 1981).

Mugineic acid (MA) is the most common and the first identified phytosiderophore (Takemoto *et al*., 1978) (Fig. 1). The stability constant of the MA-Fe(III) complex is K = 10^20^ (Raymond *et al*., 1984), which is low compared with the stability constant of microbial siderophores such as ferrichrome (K = 10^29^), ferrioxamine B (K = 10^31^) and enterobactin (K = 10^52^) (Schwarzenbach and Schwarzenbach, 1963; Harris *et al*., 1979). Other types of phytosiderophores have also been isolated from gramineous plants such as avenic acid A from oats (*Avena sativa*) and distichonic acid from beer barley (*Hordeum vulgate*) (Nomoto *et al*., 1981). In general, several studies showed that plant species such as barley, rye and wheat, which produce a high concentration of phytosiderophores, are more resistant to Fe deficiency than other species such as maize, sorghum and rice, which produce a lower concentration of phytosiderophores (e.g. Masuda *et al*., 2009; Kobayashi *et al*., 2010).

## Role of siderophores in nature

### Soil mineral weathering

In soils, the microbial communities that colonize mineral surfaces differ from the inhabitants of the surrounding soil (Certini *et al*., 2004). Microbial attachment to mineral surfaces leads to the formation of a microenvironment that protects the microorganisms against environmental stresses (Liermann *et al*., 2000; Ojeda *et al*., 2006). In these microenvironments, mineral nutrients can be chelated directly from the soil minerals or shared amongst the surrounding microorganisms (Roberts Rogers and Bennett, 2004). Siderophores produced by soil microorganisms can promote the mineral dissolution of insoluble phases (Kraemer, 2004; Shirvani and Nourbakhsh, 2010). There are different mechanisms suggested for siderophore-promoted Fe dissolution (see Holmén and Casey, 1996). The general mechanism is that the Fe(III)–siderophore complex is formed at the mineral surface and is then transferred into the surrounding soil solution and becomes available for uptake by microorganisms or plants (Kalinowski *et al*., 2000; Kraemer, 2004).

The impact of siderophores on soil mineral weathering can be more effective compared with that of low molecular mass organic acids (LMMOAs) because siderophores form more stable complexes with Fe. Siderophores form 1:1 complexes with Fe(III), with constants ranging between K = 10^30^ and K = 10^52^ (Matzanke, 1991), while the constants of oxalic and citric acids with Fe(III) are K = 10^8^ and 10^12^ respectively (Perrin, 1979). However, when both LMMOAs and siderophores are present, they may function synergistically, which results in a higher mineral dissolution rate compared with siderophore only (Reichard *et al*., 2007).

Because of the importance of microbial siderophores in weathering and soil formation, the role of siderophores in the dissolution of Fe minerals has been investigated intensively. For instance, Watteau and Berthelin (1994) reported that hydroxamate siderophores produced by *Suillus granulatus* have a high efficiency in the dissolution of goethite. Significant quantities (10^−9^ mol m^−2^ h^−1^) of Fe were mobilized in the presence of *Suillus* sp. because of its continuous production of siderophores. In addition, fungal siderophores such as ferrichrome and ferricrocin belonging to the ferrichrome family contributed in changing the surface structure of biotite and increasing its dissolution in podzolic forest soil (Sokolova *et al*., 2010). The dissolution of Fe from hornblende has also been observed to be higher in the presence of siderophore-producing actinobacteria (*Streptomyces* and *Arthrobacter*) compared with the dissolution of Fe by a synthetic siderophore (desferrioxamine B) (Kalinowski *et al*., 2000).

Some studies have also shown that phytosiderophores can play a significant role in mineral-weathering processes by accelerating the dissolution of some Fe-containing minerals such as ferrihydrate and goethite (Hiradate and Inoue, 2010). Reichard and colleagues (2005) reported that the maximum dissolution rate of goethite (1.7 nmol m^−2^ h^−1^) in the presence of phytosiderophores (2′-deoxymugineic acid) was found at pH 6. Recently, genetic engineering applications have allowed us to observe that the expression of Fe–phytosiderophore transporter genes in barley enhances its ability to dissolve Fe from soil minerals (Gómez-Galera *et al*., 2012).

### Biogeochemical cycling of Fe in the ocean

The biogeochemical cycling of trace metals in the oceans has become a subject of great concern. Of all trace metals present in the marine waters, Fe have received the most attention because it is an essential micronutrient for marine organisms, in addition to its low concentration in the ocean controls the productivity and community structure of phytoplankton (Gledhill and Buck, 2012). Marine bacteria produce most of the organic Fe chelators present in seawater and thereby play a significant role in the biogeochemical cycling of Fe in the ocean (Boyd *et al*., 2007). Those bacteria compete with phytoplankton for Fe by producing different types of siderophores that have a great impact on the Fe abundance and solubility in the marine environment (Cordero *et al*., 2012). Marine siderophores include a hydroxyl–carboxylate functional group, provided either by citrate (i.e. snychobactins, petrobactin, aerobactin and marinobactins) or by b-hydroxyaspartate (i.e. aquachelins, loihichelins and alterobactin) (Martinez and Butler, 2007; Hider and Kong, 2010).

In the surface water, siderophores participate in the photochemical cycling of Fe by forming Fe(III)–siderophore complexes that increase Fe availability for phytoplankton (Barbeau *et al*., 2001; Hunter and Boyd, 2007). Ferrioxamine G was found to be widely distributed in surface waters throughout the Atlantic Ocean, whereas ferrioxamine E had a more varied distribution within depths. These findings suggest that marine siderophores are important in enhancing the Fe abundance and availability in the water column of the Atlantic Ocean, thus playing an important role in the biogeochemical cycle of Fe (Mawji *et al*., 2008; Amin *et al*., 2012).

## Biotechnological applications of siderophores

### Enhancing growth and pathogen biocontrol of plants

It is known that microbial siderophores provide plants with Fe nutrition to enhance their growth when the bioavailability of Fe is low (Crowley, 2006), whereas the exact mechanism is fairly unknown. Two possible mechanisms were suggested by which plants could obtain Fe from microbial siderophores: (i) Microbial siderophores with high redox potential can be reduced to donate Fe(II) to the transport system of the plant. In this mechanism, it has been hypothesized that the microbial Fe(III)–siderophores are transported to the apoplast of the plant root where siderophore reduction may be occur (Mengel, 1995). Consequently, Fe(II) is trapped in the apoplast, which leads to high Fe concentrations in the root (Kosegarten *et al*., 1999). (ii) Microbial siderophores can chelate Fe from soils and then do a ligand exchange with phytosiderophores (Masalha *et al*., 2000). This mechanism depends on several parameters, i.e. the stability constants and concentrations of both microbial and phytosiderophores, and the pH and redox conditions of the root environment (Crowley, 2006).

Siderophores have been suggested to be an environmentally friendly alternative to hazardous pesticides (Schenk *et al*., 2012). It has been known for more than three decades that different *Pseudomonas* species can improve plant growth by producing siderophores (pyoverdines) and/or by protecting them from pathogens, and thus this group of bacteria was classified as plant growth-promoting bacteria (Kloepper *et al*., 1980; Gamalero and Glick, 2011). In addition to pseudomonads, other bacteria such as *Azadirachta indica* which produce ferrioxamines could contribute into plant Fe nutrition and promote the root and shoot growth (Siebner-Freibach *et al*., 2003; Verma *et al*., 2011). Mycorrhizal fungi can also be used as biofertilizers to enhance plant growth. Mycorrhizal sorghum plants were shown to take up higher concentrations of Fe than nonmycorrhizal plants (Caris *et al*., 1998). It is suggested that the ectomycorrhizal fungi associations in plant nutrition depend on fungal siderophores (Van Schöll *et al*., 2008). Recently, the plant growth-promoting activities of fungi were investigated, and siderophores produced by *Aspergillus niger*, *Penicillium citrinum* and *Trichoderma harzianum* were found to increase the shoot and root lengths of chickpeas (*Cicer arietinum*) (Yadav *et al*., 2011).

Furthermore, the significant role of siderophores in the biological control mechanism has also been demonstrated by Kloepper and colleagues (1980). This mechanism depends on the role of siderophores as competitors for Fe in order to reduce the Fe availability for the phytopathogens (Beneduzi *et al*., 2012). There are several studies regarding the role of siderophores in the biological control of plant pathogens. Pyoverdine siderophores produced by pseudomonads were found to control the wilt diseases of potato caused by *Fusarium oxysporum* (Schippers *et al*., 1987), in addition to being involved in the biocontrol of *Gaeumannomyces graminis*, which causes a deficiency of wheat and barley growth (Voisard *et al*., 1989). Furthermore, pyoverdines were also observed to suppress the phytopathogens in peanuts and maize (Pal *et al*., 2001). There are other bacterial species besides pseudomonads that can be used as biocontrol agents. For example, siderophores produced by *Bacillus subtilis* had a significant role in the biocontrol of *F. oxysporum*, which causes the *Fusarium* wilt of pepper (Yu *et al*., 2011). Also, siderophores produced by *A. indica* had a high affinity to chelate Fe(III) from soil and thereby negatively affect the growth of several fungal pathogens (Verma *et al*., 2011).

### Biocontrol of fish pathogens

Siderophores play an important role in disease control of fish by limiting Fe that is necessary in virulence and bacterial interactions (Li and Chi, 2004). The pathogenic bacterium use two different ways in infecting the fish host: (i) by production of harmful enzymes such as proteases and cholesterol acyl transferase to resist the defense mechanisms of the host (Ellis, 1999) and (ii) by production of transferrin in order to compete with the host for Fe and suppress its growth (Yano, 1996). The biocontrol mechanism mainly depend on the competition between the transferrin produced by pathogens and the siderophore produced by biocontrol agents in forming complexes with Fe, and the siderophore is always the winner because of its much higher Fe stability constants (Gram *et al*., 1999). Siderophore-producing bacteria (*Pseudomonas fluorescens*) can inhibit the growth of several fish pathogens (*Vibrio anguillarum*, *Vibrio ordalii, Aeromonas salmonicida*, *Lactococcus garvieae, Streptococcus iniae, Flavobacterium psychrophilum* and *c ruckeri*) and is therefore used as probiotics in fish farming (Gram *et al*., 2001; Brunt *et al*., 2007; Dimitroglou *et al*., 2011). Furthermore, *Bacillus* sp. has also been recommended to be used as a biocontrol agent in fish intestines and culture water (Sugita *et al*., 1998). For instance, *Bacillus* sp. strain NM 12 produces siderophores with a wide antibacterial spectrum that inhibited the growth of 62.5% of 363 intestinal bacteria identified from several coastal fishes (Li and Chi, 2004). In addition, siderophores produced by *Bacillus cereus* has also been found to inhibit the growth of the fish pathogen *Aeromonas hydrophila* (Lalloo *et al*., 2010). Recently it was found that several siderophore-producing bacteria isolated from the intestinal tracts of fishes inhibit fish pathogens such as *Aliivibrio logei*, *Vibrio ichthyoenteri*, *V. anguillarum, Vibrio splendidus* and *A. salmonicida* (Sugita *et al*., 2012).

### Microbial ecology and taxonomy

Siderotyping is defined as the characterization of microbial strains according to the siderophore types they produce (Neilands, 1981; Bosne and Levy Frebault, 1992). There are two different methods for siderotyping, the analytical and the biological methods (Meyer *et al*., 2002). The analytical methods using high-performance liquid chromatography (HPLC) coupled with mass spectrometry are based on the physicochemical properties of siderophores. However, the biological methods are based on the direct measurement of siderophore-mediated Fe in the microbial cells or using a molecular biology method based on the recognition of specific DNA sequences related to siderophores (Bach *et al*., 2000).

*Pseudomonas*, produce mainly over 50 different pyoverdine siderophores (Boukhalfa and Crumbliss, 2002), in addition to a wide variety of other siderophore types (Cornelis, 2010). The peptide chain of the fluorescent pyoverdine varies among the different species and this variability can be easily used to determine the relatedness of these species (Chincholkar *et al*., 2005). Siderotyping application has been previously investigated on 400 strains of fluorescent and non-fluorescent *Pseudomonas* spp. and these strains were grouped into 28 taxa including 15 well-defined species depending on the different siderophore types (Meyer *et al*., 2002). Sixty-eight fluorescent *Pseudomonas* strains were also identified using mass spectrometry analysis of their pyoverdines, and thus siderotyping was recommended as a helpful method for studying microbial diversity and taxonomy (Meyer *et al*., 2008; Meyer, 2010). Other studies have found that siderophore production can be used as a chemotaxonomic marker for the identification of other types of bacteria such as *Burkholderia* spp. and *Mycobacterium* spp. based on the variation of the chemical structures of ornibactins and mycobactin respectively (Mokracka *et al*., 2004; Bultreys *et al*., 2006). Those findings suggest that siderotyping could become a powerful tool in environmental research because it can provide a quick and unambiguous identification of microbes at the species level (Meyer *et al*., 2002).

### Bioremediation of environmental pollutants

#### Metals

Metals play a vital role in the development of human civilizations (Jonhson *et al*., 2002), but the manufacturing industry, sludge applications, nuclear power stations and mining have led to metal pollution (Wasi *et al*., 2013). Siderophores are extremely effective in solubilizing and increasing the mobility of a wide range of metals such as Cd, Cu, Ni, Pb, Zn, and the actinides Th(IV), U(IV) and Pu(IV) (Schalk *et al*., 2011). This ability of siderophores mainly depends on their ligand functionalities, by which means siderophores may have a strong affinity or selectivity for a particular metal other than Fe with regards to the stability constants of this metal–siderophore complex (Hernlem *et al*., 1999). Thereby, siderophores become a useful tool in bioremediation, which is a cost-effective and environmentally friendly technique (Rajkumar *et al*., 2010).

During the last years, there had been an increasing interest in investigating the potential of using siderophores in metal bioremediation. Neubauer and colleagues (2000) showed that siderophores such as desferrioxamine B could chelate Co(III) better than Fe(III) in high pH conditions. *Azotobacter vinelandii*, which produced siderophores, i.e. azotochelin and azotobactin, had the ability to use those siderophores for both Mo and V acquisition (Wichard *et al*., 2009). Moreover, the siderophore pyochelin produced by *Pseudomonas aeruginosa* could chelate a wide range of metals, i.e. Ag^+^, Al^3+^, Cd^2+^, Co^2+^, Cr^2+^, Cu^2+^, Eu^3+^, Ga^3+^, Hg^2+^, Mn^2+^, Ni^2+^, Pb^2+^, Sn^2+^, Tb^3+^, Tl^+^ and Zn^2+^; however, the uptake process did not appear to assimilate any metal other than Fe^3+^ (Braud *et al*., 2009a). Hong and colleagues (1996) reported that siderophores produced by *Fusarium solani* contributed in solubilizing Cu and Zn in vitro. Siderophores also played a significant role in mobilizing metals from mine waste material or metal-contaminated soils. Several metals (i.e. Fe, Ni and Co) were mobilized from waste material (acid-leached ore) of a former uranium mine in the presence of siderophores produced by *P. fluorescens* (Edberg *et al*., 2010). It has been shown that the siderophores produced by *Agrobacterium radiobacter* removed approximately 54% of the As from a metal-contaminated soil (Wang *et al*., 2011). Recently, it has been found that pyoverdines mobilized U(VI), Np(V) and other metals from uranium mine waste (Behrends *et al*., 2012).

Not only microbial siderophores contribute to metal bioremediation, but also there are many studies that have demonstrated that phytosiderophores are efficient in mobilizing metals in soil (e.g. Rajkumar *et al*., 2009; 2010; 2012). Phytosiderophores have a high affinity for complexation with several metals in the following order (Cd^2+^ > Ni^2+^ > Pb^2+^ > Sn^2+^ > AsO_4_^−2^ > AsO_2_^−1^ > Mn^2+^ > Co^2+^ > Cu^2+^ > Fe^+3^) and very weakly binds Al^3+^ and Cr^3+^ (Ruggiero *et al*., 1999). Studies in both uncontaminated and contaminated soils have shown that phytosiderophores are more efficient in mobilizing Fe, Cu, Zn, Ni and Cd from the soil in comparison with synthetic chelators and microbial siderophores (i.e. Awad and Römheld, 2000; Singh *et al*., 2008).

#### Petroleum hydrocarbons

Petroleum hydrocarbons in marine ecosystems are one of the major environmental problems. Microorganisms could play an important role in the remediation of petroleum hydrocarbons from the marine environment (Das and Chandran, 2011). Microbial siderophores participate in the biodegradation of petroleum hydrocarbons through an indirect mechanism, by facilitating the Fe acquisition for the degraded microorganisms under Fe-limiting conditions (Barbeau *et al*., 2002). Petrobactin was the first structurally characterized siderophore produced by the oil-degrading marine bacterium *Marinobacter hydrocarbonoclasticus* (Barbeau *et al*., 2002). Hickford and colleagues (2004) identified another sulfonated siderophore called ‘Petrobactin sulfonate’ isolated from the same oil-degrading marine bacterium. Few studies suggested that the use of siderophores may be a good strategy for oil spill clean-up. Gauglitz and colleagues (2012) showed recently that marine *Vibrio* spp. isolated from the Gulf of Mexico after the 2010 Deepwater Horizon oil spill, produce amphiphilic siderophores called ‘ochrobactins’ that were suggested to efficiently contribute in the degradation of petroleum hydrocarbons.

### Nuclear fuel reprocessing

Siderophores contain anionic hydroxamate or catecholate functional groups that form hard oxodonors that strongly bind to Lewis acids, resulting in complexes with remarkably high stability constants (Harris *et al*., 1979). Because actinides form strong complexes with hard oxygen anions, it has been suggested that siderophores could bind actinides with a complexation constant estimated to be K = 10^16^ (Raymond, 1990; Jarvis and Hancock, 1991).

The Purex process has been used commercially to reprocess irradiated nuclear fuel by solvent extraction techniques and separate U and Pu for reuse from fission products such as Ti and Np (Taylor and May, 1999). During this process, U and Pu flow into the solvent and become contaminated with Np. Siderophores have been shown to allow for the selective removal of Np from the solvent phase (Taylor *et al*., 1998), and thus siderophores could be used in the Purex process to simplify the removing of the actinides (Renshaw *et al*., 2002). Desferrioxamine B forms a stable complex with U(VI), where its hydroxamate functional group is similar to acetohydroxamic acid, a ligand that has been proposed for actinide complexation (Mullen *et al*., 2007). Recently, Marshall and colleagues (2010) reported that low concentration of siderophores was enough to potentially influence the dissolution of spent nuclear fuel, and it seemed not to be a significant difference between using the synthetic desferrioxamine B or pyoverdine produced by *P. fluorescens*. Based on those studies, siderophores have been proposed for the remediation of radioactive waste and reprocessing of nuclear fuel.

### Optical biosensor

A biosensor is a biomolecule coupled to an electrical device such as a transducer, amplifier or noise filter in order to increase the signal to noise ratio that allows detection of various types of responses through specifically engineered systems (Gupta *et al*., 2008). Pyoverdines are yellow-green water-soluble fluorescent siderophores characterized by the following properties (Barrero *et al*., 1993): (i) They form a strong complex with Fe(III) and have a weak or negligible affinity for Fe(II), and (ii) the Fe(III) complexes have very high stability constants (approximately K = 10^32^) (Kurtz and Crouch, 1991). These characteristics make pyoverdine a promising agent for the construction of optical biosensors (Pesce and Kaplan, 1990). Using the siderophore with an exceptional Fe(III)-binding constant would be an ideal choice for the molecular recognition element of the sensor that could be applied in the determination of Fe bioavailability in oceanic water or soils (Chung Chun Lam *et al*., 2006). The concentration of Fe present in the ocean has been determined by using a siderophore as a biosensor (Chung Chun Lam *et al*., 2006). In that study, they used parabactin that was produced by *Paracoccus denitrificans* as a biosensor by encapsulating it in sol-gel thin film on a quartz substrate. The seawater samples were analysed by a flow cell that was mounted in the sample partition of the fluorescence spectrometer. Siderophores also provide the potential for a good sensitive and selective detection system that would mimic the biological uptake process (Ellerby *et al*., 1992). For instance, azotobactin produced by *A. vinelandii* has been used as an optical biosensor for Fe(III) in a modified design that depends on the encapsulation of the azotobactin in sol-gel matrices without significant loss of its fluorescence signal (Sharma and Gohil, 2010). Additionally, the Fe(II and III) specificity for the Fe biosensor pyoverdine has been optimized by immobilizing it in three formulations of porous sol-gel glass (A, B and C), which contained various amounts of water added (Yoder and Kisaalita, 2011). In that study, the most specific and linear response for binding to Fe(II and III) was observed for pyoverdine immobilized in sol-gel C that had more water than A and B.

### Bio-bleaching of pulps

The pulp and paper industry is a primary source of many environmental problems including global warming, human toxicity, ecotoxicity, photochemical oxidation, acidification, nitrification and solid wastes (Singh *et al*., 2008; Bajpai, 2010). The main problem of pulp and paper manufacturing results from the bleaching processes. Some pollutants are emitted into the air, while others are discharged in wastewater.

Siderophores are considered as effective agents in pulp treatment, where they can reduce 70% of the chemicals needed to bleach Kraft pulp (Bajpai, 2004), and that makes siderophores environment-friendly alternatives. Brown-rot fungi are considered to be one of the most important groups of wood-decaying microorganisms. Some studies reported the production of catecholate and hydroxamate siderophores by wood-decaying fungi (Fekete *et al*., 1989; Fekete, 1993). For instance, hydroxamate siderophores isolated from the brown-rot fungus (*Gloephyllum trabeum*) had the ability to mediate the reduction of Fe in redox cycling processes. The reduced Fe can then react with hydrogen peroxide to generate oxygen radical species that depolymerize cellulose, hemicellulose and lignocellulose. This depolymerization process was considered to be the main role of siderophores in the bio-bleaching of pulps (Xu and Goodell, 2001; Arantes and Milagres, 2007). Moreover, Milagres and colleagues (2002) showed the ability of siderophores produced by different microbial species in bio-bleaching of pulps with regard to the loss of viscosity and they found that siderophores produced by *G. trabeum* could degrade pulp with 10.8% loss of viscosity, whereas siderophores produced by *Perenniporia medula-panis* and *Tinea versicolor* degraded pulps with 13.6% and 14.4% loss respectively. It has also been observed that siderophores produced by *Coriolus versicolor* could alter the lignin structure to make it more susceptible to degradation and accordingly contribute in bio-bleaching of pulps (Wang *et al*., 2008).

## Concluding remarks and future perspectives

The study of mineral–microbe interactions underscores the importance of microorganisms in making the earth a suitable environment for all forms of life. In recent years, it has become clear that siderophores represent central organic compounds in Fe uptake in many microorganisms and plants. Understanding the chemical structures of different siderophores and the membrane receptors involved in Fe uptake has opened new areas for research. The importance of siderophores is obvious, and they play a significant role in the environmental applications, even if there are many questions remaining to be answered. What is the specific role of microorganisms and plants in the selectivity of metal uptake by siderophores? Why do microorganisms secrete more than one type of siderophores to meet their mineral nutritional needs? What is the relative importance of the different siderophore structures involved in environmental applications? Can modified genetic methods such as labelled DNA be useful tools for the direct detection of siderophore functional genes in the environment?

More research is focusing on finding effective ways to use siderophores in bioremediation and biocontrol, which should enhance their application in the environment. Siderophore variability and their structural and functional characteristics in relation to microbial communities must be vigorously investigated to improve the role of siderophores in environmental applications. The relationship between siderophores and microbial structure, in environments with low Fe bioavailability, i.e. oceans and some soil conditions, are still not clearly known. Combining metagenomics with detailed chemical analysis will reveal important information that could be used to improve the current environmental applications and develop new applications for siderophores.
